# Acute and sub-acute toxicity of ethanol extracts of *Hagenia abyssinica* and *Rumex abyssinicus* flowers in Swiss albino mice

**DOI:** 10.1371/journal.pone.0319464

**Published:** 2025-02-25

**Authors:** Hirut Basha Gemeda, Asfaw Debella, Milkyas Endale, Abiy Abebe, Meharu Mathewos, Wossene Habtu, Dinkenesh Chalchisa, Betelhem Getachew, Menal Hassen, Hassen Mamo

**Affiliations:** 1 Traditional and Modern Medicine Research and Development Directorate, Armauer Hansen Research Institute, Addis Ababa, Ethiopia; 2 Clinical Chemistry laboratory Unit, Ethiopian Public Health Institute, Addis Ababa, Ethiopia; 3 Hematology laboratory Unit, Ethiopian Public Health Institute, Addis Ababa, Ethiopia; 4 Pathology Laboratory Unit Armauer Hansen Research Institute, Addis Ababa, Ethiopia; 5 Department of Microbial, Cellular and Molecular Biology, College of Natural and Computational Sciences, Addis Ababa University, Addis Ababa, Ethiopia; Dr Anjali Chatterji Regional Research Institute for Homoeopathy, INDIA

## Abstract

**Background:**

*Hagenia abyssinica* (Bruce) J.F. Gmel (Family: Rosaceae) and *Rumex abyssinicus* Jacq (Family: Polygonaceae) are valuable medicinal plants traditionally used in Ethiopia to treat various diseases. Recent studies have also demonstrated that solvent extracts of these plants exhibit molluscicidal activities under laboratory conditions, highlighting their potential for snail control. However, limited information is available regarding their safety profiles.

**Objective:**

This study aimed to evaluate acute, and sub-acute toxicity of 70% ethanol extracts of *H. abyssinica* and *R. abyssinicus* flowers in Swiss albino mice, following the Organization for Economic Co-operation and Development guidelines 423 and 407.

**Methods:**

In the acute toxicity study, both extracts were administered orally to experimental groups at varying concentrations (mg/kg bodyweight): 5, 50, 300, and 2000. For the sub-acute toxicity study, both extracts were given to the experimental groups at doses (mg/kg) of 125, 250, and 500 daily for 28 days. Blood samples were collected from each mouse and analyzed for hematological and biochemical parameters. Additionally, the heart, liver, and kidneys were excised, stained, and examined for potential histopathological effects.

**Results:**

The acute toxicity study revealed no noticeable changes in behavior at the highest oral dosage of 2000 mg/kg*.* In the sub-acute toxicity study, no statistically significant changes were observed in hematological and biochemical parameters compared to the control group. Similarly, no abnormal histological findings were noted in the examined organs in comparison to the control group*.*

**Conclusion:**

These findings indicate that flower extracts of both plants did not show significant toxicity to laboratory mammals at an oral dosage of 2000 mg/kg.

## Introduction

Schistosomiasis is a leading helminthic disease caused by trematode worms of the genus *Schistosoma,* phylum: Platyhelminthes [1,2]. While it occurs worldwide, it is particularly prevalent in Africa, South America, the Caribbean Islands, and the Eastern Mediterranean [3,4]. In 2022, there are an estimated 264 million individuals globally in need of preventive chemotherapy for schistosomiasis, with 91.3% of them residing in Africa [5]. In Ethiopia, an estimated 53.3 million population was at risk for schistosomiasis in 480 districts [6]. *S. mansoni* and *S. haematobium*, transmitted by *Biomophlaria* and *Bulinus* snails, respectively, are endemic in Ethiopia [7]. *S. mansoni* is widespread across many parts of the country, while *S. haematobium* is found in a few isolated areas [8].

The new World Health Organization Neglected Tropical Diseases Roadmap for 2021–2030 recommends the elimination of schistosomiasis as a public health problem and the interruption of transmission in humans by 2030 [9]. In line with this, Ethiopia has developed a strategic plan for schistosomiasis elimination to stop the transmission of the disease by 2030 [6]. To achieve this goal, various strategies are being implemented including early diagnosis and treatment, health education, provision of safe and adequate water supply, increasing sanitation coverage, and targeting snail intermediate hosts [6]. Each of these approaches faces its own real-world challenges. For instance, the drug praziquantel (PZQ) is effective against all types of schistosome species and can kill the worms within a few hours [10]. However, in countries where schistosomiasis is common, especially in low-income countries, it is not feasible to treat a large number of people due to the high overall cost [11]. Furthermore, parasite resistance to PZQ raises concerns about its future effectiveness [12–15]. Health education is important for changing behaviors such as stopping open defecation and urination, and reducing contact with water infested with schistosomiasis [16]. However, behavioral change alone is not enough to control schistosomiasis; it must be accompanied by providing sanitation and a safe, adequate water supply [11]. Similarly, improving sanitation, housing conditions, and increasing the coverage of safe and adequate water supply are the best strategies for preventing and controlling the transmission of schistosomiasis [17]. However, these strategies are not feasible or cost-effective in the near future, especially in low-income countries where schistosomiasis prevalence is high, as they require significant investment and government commitment. Eliminating snails using chemical molluscicides is a common practice for controlling the transmission of schistosomiasis [18,19] and can be scaled-up and integrated into other control activities. However, the use of chemical molluscicides has several limitations: they can be toxic to non-target species, are often not cost-effective, and pose environmental risks [20–22]. In contrast, effective and less-toxic medicinal plants have the potential to be used as more cost-effective, and environmentally friendly tools to controlling schistosome snails.

*Hagenia abyssinica* (Bruce) J.F. Gmel., known as *kosso* in Amharic and belonging to the Rosaceae family, has been a key medicinal plant used since ancient times to treat various ailments including intestinal parasites, especially against cestodes [23,24]. This species is found across Africa, particularly in Kenya, Tanzania, Uganda, Sudan, Congo, Malawi, Burundi, Rwanda, and Ethiopia [25] *Rumex abyssinicus* Jacq, known as *Mekmeko* in Amharic and belonging to the Polygonaceae family, grows in the highlands of tropical Africa and is distributed across North Africa and Ethiopia [26] where it is widely used as a traditional medicine for conditions such as liver diseases, hepatitis, malaria, scabies, blood pressure, jaundice, wound and pneumonia [27,28]. In our laboratory study, 70% ethanol extracts of *H. abyssinica* flowers exhibited strong molluscicidal activity [29]. Similarly, 70% of ethanol extracts of *R. abyssinicus* flowers exhibited strong molluscicidal activity (unpublished data).

Based on these promising preliminary results, it is crucial to evaluate the safety profiles of these plants, as data on this subject are scarce. This study aimed to determine the lethal dose (LD_50_), as well as the oral acute and sub-acute toxicity of 70% ethanol extracts of the flowers of *H. abyssinica* and *R. abyssinicus* in Swiss albino mice. Evaluating the toxicological properties of these traditional medicinal plants is crucial for assessing their potential risks to local communities. Understanding their safety profiles will have multiple implications, including guiding their use against schistosome snail intermediate hosts while minimizing impacts on non-target aquatic organisms. Ultimately, these toxicological studies will contribute to the responsible application of these plants in traditional medicine and their potential role as eco-friendly molluscicides for schistosomiasis control.

## Materials and methods

### Plant materials

Female flowers of *H. abyssinica* and *R. abyssinicus* were collected in 2023 from the *Gulele Botanical Garden* in Addis Ababa, Ethiopia. This garden is located between latitudes 8°55’N and 9°05’N and longitudes 38°05’E and 39°05’E. Mr. Melaku Wondafrash, a botanist at Addis Ababa University, confirmed the plant specimens. The National Herbarium of Addis Ababa University holds the voucher specimens with identifiers HB002 for *H. abyssinica* and HB003 for *R. abyssinicus*

### Extraction of plant materials

The solvent used to extract the plant’s materials was 70% ethanol (Loba Chemie India). The grounded plant materials were macerated with 70% ethanol while continuously shaking on an orbital shaker (Benchmark Scientific, Inc. USA) for 24 hours to maximize extraction. Each extract was filtered by using Whatman no.1 (Whatman International Ltd., England) filter paper the residue was re-macerated three times. Solvents were removed using a rota evaporator at 40^0^C to concentrate the crude extract (BUCHI Labortechnik AG, Switzerland). The dried extracts were transferred to a screw-capped glass container and kept in the refrigerator until used for the experiment [30]. The yield of the extracts was 11.4g from *H. abyssinica* flowers and 16.0 g from *R. abyssinicus.*

### Experimental animals and ethical considerations

A total of 110, Swiss albino mice (*Mus musculus*) of both sexes (25–30g), 8 weeks old were obtained from the animal breeding unit of the Ethiopian Public Health Institute (EPHI). Of these mice, 30 were used in the acute toxicity test and 80 for the sub-acute toxicity test. For the acute toxicity study, only female nulliparous and non-pregnant mice were used. However, both sexes were used for the sub-acute toxicity test. The experimental animals were kept under standard laboratory conditions temperature and humidity and light (12 h light and 12 h dark) cycle. They were fed with pellets, which are the standard diet, and water *ad libitum*, and the animals were acclimatized at the laboratory for five days. All animals involved in this study were treated humanely throughout the study period in accordance with the International Guidelines of Laboratory Animal Care and Use and The European Union Guidelines Directive 2010/63/EU for the housing, care, and use of the experimental animals [31,32]. For euthanasia, the American Veterinary Medical Association (AVMA) guidelines were used [33,34]. The experimental procedures received approval from the Ethiopian Public Health Institute Review Board Committee on July 24, 2023. The protocol number is EPHI-IRB-513-2023, and the reference number is EPHI6.13/83. Finally, the carcasses of the sacrificed animals were disposed of humanely following the AVMA guidelines [33]. This study was performed in accordance with the ARRIVE guidelines for reporting animal research [35].

### Acute oral toxicity study

An acute oral toxicity study of the 70% ethanol extracts of the plants was conducted according to the Organization for Economic Co-operation and Development (OECD) guidelines 423 [36]. Thirty female mice, aged 8 weeks, were divided into ten groups, with three mice in each group. The first five groups were assigned to the *H. abyssinica* experiment, while the remaining five groups were assigned to the *R. abyssinicus* experiment.

Group I and Group VI served as the controls for *H. abyssinica* and *R. abyssinicus*, respectively, and received distilled water as a vehicle. Group II to Group V and Group VII to Group X received a single dose *H. abyssinica* and *R. abyssinicus* extracts at varying concentrations (mg/kg): 5, 50, 300, and 2000, respectively, after fasting for three to four hours. The extract was delivered using an oral gavage feeding needle. Following administration, food was withheld for an additional one to two hours.

The animals were then individually observed for signs of toxicity at least once during the first 30 min after dosing, periodically during the first 24 h, and daily thereafter, for a total of 14 days to monitor and record any behavioral indicators of toxicity, including piloerection, debilitation, tremors, excitability, salivation, twitching, diarrhea, and lethargy [33,34].

### Sub-acute toxicity study

Sub-acute toxicity studies were conducted using male and female mice. A total of 40 male and 40 female mice were used, with 20 male and 20 female, all aged 8 weeks, for each extract experiment. For *H. abyssinica*, the mice were divided into eight groups of five mice each randomly. Groups I to IV consisted of female mice, while Groups V to VIII consisted of male mice. Similarly, for *R. abyssinicus*, the mice were divided into eight groups of five mice each. Groups IX to XII consisted of female mice, while Groups XIII to XVI consisted of male mice. Groups I and V, as well as Groups IX and XIII, served as the controls for female and male *H. abyssinica* and female and male *R. abyssinicus*, respectively, and received distilled water as a vehicle. Groups II to IV and VI to VIII were given daily doses of the 70% ethanol extract of *H. abyssinica* flowers at varying concentrations (125, 250, and 500 mg/kg) for 28 days via oral gavage. Similarly, Groups X to XII and XIV to XVI were given daily doses of the 70% ethanol extract of *R. abyssinicus* flowers at varying concentrations (125, 250, and 500 mg/kg) for 28 days via oral gavage.

The dose level for the sub-acute toxicity study was administered based on the acute toxicity results and OECD guidelines 407 [37]. The dosing volume was set at 1 mL per 100 g of body weight. Throughout the experiment, body weight, water intake, food consumption, behavioral parameters, and any signs of toxicity were observed and recorded daily. At the end of the treatment, the animals were fasted overnight prior to euthanasia and anesthetized by pentobarbital injection (50 mg/kg body weight), and then blood was collected via cardiac puncture for hematological and biochemical analysis.

### Weekly bodyweight and relative organ weight

The bodyweight of each mouse was measured using a sensitive balance (RADWAG, UK) during the acclimatization period, once before the start of dosing, weekly during the dosing period, and on the day of sacrifice. On the 29th day, after fasting overnight, the animals were sacrificed humanely using an overdose of anesthesia. The liver, kidneys, and heart were carefully dissected [38] and weighed in grams. The relative organ weight for each animal was then calculated using the equation as follows according to previously published methods [39,40]: Relative organ weight (%) =  100 ×  absolute organ weight (g)/bodyweight (g).

### Biological specimen collection

On the 29th day, the animals were anesthetized and blood samples were collected via cardiac puncture using a gauge needle attached to a 5 ml syringe. Blood samples were taken from all animals into tubes with and without the anticoagulant Ethylene Diamine Tetra-Acetic Acid (EDTA) for hematological and biochemical tests, respectively.

### Hematology

One ml of blood collected in a plastic test tube containing EDTA was used to assess the hematological parameters. The blood samples were transported to the hematology laboratory (EPHI) within 30 minutes of collection, ensuring they were not exposed to freezing or excessive heat, and the samples were analyzed within 2 hours of collection. The hematological parameters include white blood cell (WBC, 10^3^/μL), red blood cell (RBC, 10^6^/μL), hemoglobin (Hb, g/dL), hematocrit (HCT %), mean corpuscular volume (MCV, fl,), mean corpuscular hemoglobin (MCH, fl,), mean corpuscular hemoglobin concentration (MCHC, g/dL), red cell distribution (RDW,%), red cell distribution width standard deviation (RDW-SD, fl)), platelet (PLT, 10^3^/μL), mean platelet volume (MPV, fl), neutrophils (NE, 10^3^/μL), lymphocytes (LY, 10^3^/μL), monocytes (MO, 10^3^/μL), eosinophils (EO, 10^3^/μL), and basophils (BA, 10^3^/μL). An automated Hematology Analyzer (Beckman Coulter hematology analyzer, Unicel DXh800 Germany) was used to analyze the samples.

### Biochemical analysis

For biochemical analysis, blood samples were collected in test tubes without anticoagulants. The clotted blood was then centrifuged within 30 minutes of collection to obtain the serum, which was stored at -20°C until assayed for biochemical analysis. The following tests were performed: liver enzymes, including alkaline phosphatase (ALP), aspartate aminotransferase (AST), and alanine aminotransferase (ALT), to assess liver toxicity; renal function indicators such as creatinine and urea; a lipid profile including total cholesterol, Low-Density Lipoprotein (LDL), High-Density Lipoprotein (HDL), and triglycerides; and glucose levels. Values in the sera were analyzed using an Automated Clinical Chemistry Analyzer (Cobas, 6000 machine, Germany).

### Histopathological examinations

Histopathological examinations were conducted on liver, kidney, and heart tissues from the experimental animals. On the 29th day, all mice from both the experimental and control groups were sacrificed humanely using an overdose of pentobarbital injection (150 mg/kg).

The liver, kidneys, and heart were then examined macroscopically for any gross pathological changes resulting from exposure to the test extracts, compared to the control groups. Tissue samples were preserved in a 10% buffered neutral formalin solution and subsequently embedded in paraffin wax. A rotary microtome (LEICA RM2255 Germany) was used to slice the sections to a thickness of 5 μm. Tissue sections were placed on glass slides and stained with hematoxylin and eosin to examine any histopathological changes [41]. All areas of the tissue morphology were observed using a light microscope at 100xmagnification, and images were captured with a digital camera attached to the microscope. The microscopic examination of the prepared slides was conducted by experienced pathologist Dr. Menal Hassen from the Amauer Hansen Research Institute (AHRI), Ethiopia. To ensure unbiased evaluation, the pathologist was blinded to the sample identities, and the samples were coded prior to the examination to prevent bias related to knowledge of group assignments.

### Statistical analysis

Data analysis was performed by the Statistical software SPSS version 24, (IBM Corp). The results were expressed as mean ±  SD (standard deviation of the mean). One-way ANOVA with Dunnett’s multiple comparisons test was performed to compare the means between the control and experimental groups. The values were considered significantly different from the control group for the treatment groups when p <  0.05.

### Quality control

To ensure the validity and reliability of this study, we implemented several quality control measures. Firstly, we selected healthy 8-week-old mice within an appropriate weight range to ensure consistency across groups. The animals were randomly assigned to control and treatment groups to avoid bias. Additionally, the animals were given time to acclimate to the laboratory environment for a period of 5-7 days before the study began. The blood samples were transported to the laboratory within 30 minutes and stored at a temperature of 2-8°C until processing. The stained slides for histopathological examination were stored in appropriate containers to protect them from prolonged exposure to light.

Furthermore, the dosage of the plant extracts was administered according to each animal’s bodyweight. All observations, measurements, and test results were consistently and systematically recorded and labeled properly to avoid sample mix-ups and misidentification. The study adhered to good laboratory practices (GLP) and relevant regulatory standards, such as OECD 423 and 407 guidelines. Blinding was implemented whenever necessary to ensure unbiased evaluation. The data were entered in Excel 2010 and later imported into SPSS version 24 for data cleaning and analysis.

## Results

### Acute toxicity

In the acute toxicity experiment, the test animals did not show any signs of toxicity or noticeable changes in behavior when administered the highest oral dosage of 2000 mg/kg of both extract types. The bodyweight gain of the control and treatment groups was comparable. Additionally, no deaths occurred during the 14-day observation period. These findings demonstrate that the LD_50_ of the plant extracts was greater than 2000 mg/kg of body weight.

### Sub-acute toxicity and bodyweight

The bodyweight of the mice in both the control-treated groups increased over time (Table 1). For both groups treated with *H. abyssinica* and *R. abyssinicus,* both male and female mice exhibited normal patterns of bodyweight increase. The analysis revealed no significant difference in the mean bodyweights of male and female mice treated with different doses of 70% ethanol extracts of both plants compared to their respective control groups (Table 1).

**Table 1:** Effects of 70% ethanol extracts of *H. abyssinica* and *R. abyssinicus* fruits on bodyweight of Swiss albino mice.

Plant extracts	Group	Bodyweight (g)				
***H. abyssinica***		**Day 1**	**Week I**	**Week II**	**Week III**	**Week IV**
	***Male***					
	Control	29.42 ± 0.60	31.14 ± 0.65	32.28 ± 0.46	33.18 ± 0.56	34.46 ± 0.33
	125	29.38 ± 0.67	30.62 ± 0.46	31.92 ± 0.42	33.14 ± 0.39	34.50 ± 0.35
	250	29.30 ± 0.59	30.68 ± 0.66	31.72 ± 0.53	33.02 ± 0.42	34.30 ± 0.40
	500	29.42 ± 0.40	30.50 ± 0.51	31.76 ± 0.46	32.96 ± 0.24	34.34 ± 0.39
	**Female**					
	Control	26.36 ± 0.40	27.44 ± 0.67	28.08 ± 0.51	28.96 ± 0.65	29.58 ± 0.54
	125	25.82 ± 0.49	26.64 ± 0.56	27.72 ± 0.71	28.46 ± 0.68	29.54 ± 0.70
	250	26.32 ± 0.66	27.08 ± 0.58	27.96 ± 0.50	28.78 ± 0.48	29.72 ± 0.39
	500	26.34 ± 0.51	27.14 ± 0.52	28.12 ± 0.47	28.92 ± 0.30	29.88 ± 0.43
***R. abyssinicus***	**Male**					
	Control	29.18 ± 0.49	30.54 ± 0.52	31.74 ± 0.32	32.94 ± 0.40	34.28 ± 0.31
	125	29.40 ± 0.38	30.54 ± 0.37	31.72 ± 0.31	32.92 ± 0.25	34.10 ± 0.22
	250	29.04 ± 0.70	30.48 ± 0.32	31.74 ± 0.34	32.96 ± 0.30	34.18 ± 0.19
	500	28.88 ± 0.52	30.42 ± 0.44	31.60 ± 0.41	32.68 ± 0.52	33.86 ± 0.58
	***Female***					
	Control	26.28 ± 0.70	27.08 ± 0.59	27.80 ± 0.51	28.80 ± 0.63	29.78 ± 0.63
	125	26.20 ± 0.56	26.86 ± 0.48	27.84 ± 0.37	28.90 ± 0.33	30.00 ± 0.39
	250	26.40 ± 0.64	27.32 ± 0.54	28.22 ± 0.66	29.16 ± 0.69	30.04 ± 0.63
	500	26.00 ± 0.65	26.78 ± 0.63	27.62 ± 0.57	28.56 ± 0.48	29.58 ± 0.47

Note: ± values are presented as the mean ± standard deviation; the number of mice in each group is 3. There is no significant difference in bodyweight between the mice that received 70% ethanol extracts of of both plants compared to their respective control groups (p > 0.05). Extract doses are expressed in mg/kg and mice bodyweight in g.

### Vital organ relative mass

The various doses administered over 28 days did not have a significant impact on the weight of the heart, liver, and kidneys when compared to their respective control group (Table 2).

**Table 2:** Effects of 70% ethanol extracts of *H. abyssinica* and *R. abyssinicus* flower on the relative weight of the kidney, heart and liver of mice.

Group	The % of relative organ weight exposed to *Ha.*			The % of relative organ weight exposed to *Ra*		
	Kidney	Heart	Liver	Kidney	Heart	Liver
**Male**						
Control	1.53 ± 0.07	0.64 ± 0.02	6.40 ± 0.08	1.49 ± 0.05	0.62 ± 0.04	6.35 ± 0.05
125	1.53 ± 0.08	0.62 ± 0.03	6.30 ± 0.05	1.54 ± 0.06	0.61 ± 0.04	6.37 ± 0.08
250	1.51 ± 0.04	0.62 ± 0.02	6.27 ± 0.15	1.50 ± 0.03	0.60 ± 0.04	6.34 ± 0.04
500	1.50 ± 0.02	0.61 ± 0.04	6.30 ± 0.10	1.53 ± 0.05	0.63 ± 0.03	6.42 ± 0.10
**Female**						
Control	1.12 ± 0.02	0.54 ± 0.01	5.36 ± 0.09	1.15 ± 0.54	0.54 ± 0.01	5.31 ± 0.03
125	1.15 ± 0.04	0.52 ± 0.03	5.32 ± 0.09	1.12 ± 0.03	0.56 ± 0.02	5.32 ± 0.02
250	1.13 ± 0.03	0.53 ± 0.02	5.37 ± 0.06	1.11 ± 0.03	0.53 ± 0.02	5.30 ± 0.05
500	1.11 ± 0.04	0.53 ± 0.02	5.30 ± 0.08	1.12 ± 0.05	0.54 ± 0.02	5.34 ± 0.03

**Note:** ± values are presented as the mean ± standard deviation; the number of mice in each group is 3. There is no significant difference in the relative weights of the kidney, heart, and liver between the mice that received extracts of *Hagenia abyssinica* (Ha) and *Rumex abyssinicus* (Ra) compared to their respective control groups (p > 0.05). Extract doses are expressed in mg/kg.

### Hematological parameters

The hematological parameters test results for both male and female mice treated with the extract showed that all values, including WBC, RBC, Hb, HCT, MCV, MCH, RDW, RDW-SD, PLT, MPV, NE, LY, MO, and EO were within normal ranges compared to a control group. Overall, there were no significant differences in the average hematological parameters between the control and treated groups (Table 3).

**Table 3.** Effects of low- (125mg/kg), medium- (150mg/kg) and high-doses (500mg/kg) of 70% ethanol extracts of *H. abyssinica* flower on hematological parameters of mice.

Parameter	Male				Female			
	Control	Extract Dose (mg/kg)			Control	Extract Dose (mg/kg)		
		Low	Medium	High		Low	Medium	High
WBC (10^3^/μL)	6.20 ± 0.50	6.40 ± 0.56	6.30 ± 0.20	6.60 ± 0.42	5.80 ± 0.14	5.70 ± 0.28	5.60 ± 0.70	6.00 ± 0.00
RBC (10^6^/μL)	9.47 ± 0.65	9.10 ± 0.01	9.50 ± 0.70	9.89 ± 0.16	9.68 ± 0.42	9.31 ± 0.31	10.00 ± 1.40	9.91 ± 0.12
HGB (g/dL)	16.05 ± 0.21	15.00 ± 0.35	15.90 ± 0.07	15.90 ± 0.21	15.75 ± 0.63	14.95 ± 0.35	14.90 ± 1.60	15.95 ± 1.20
HCT (%)	49.25 ± 0.84	48.75 ± 1.76	48.50 ± 0.70	49.45 ± 1.62	52.05 ± 5.72	47.15 ± 1.06	46.5 ± 2.10	51.15 ± 1.06
MCV(fl)	50.05 ± 0.07	51.20 ± 1.13	51.50 ± 0.70	51.40 ± 0.56	53.70 ± 3.53	50.70 ± 0.56	50.00 ± 2.80	52.40 ± 0.56
MCH (pg)	16 ± 0.00	15.90 ± 0.42	15.5 ± 0.70	16.10 ± 0.14	15.75 ± 50.60	15.55 ± 0.70	16.00 ± 0.00	15.20 ± 0.20
MCHC (g/dl)	31.50 ± 0.70	31.05 ± 0.07	30.50 ± 0.70	31.65 ± 0.49	30.40 ± 2.12	31.65 ± 0.07	31.50 ± 2.10	29.55 ± 0.63
RDW (%)	16.50 ± 0.70	18.45 ± 1.34	18.00 ± 1.40	17.55 ± 0.63	16.35 ± 0.35	17.50 ± 0.70	18.00 ± 1.40	18.20 ± 0.28
RDW-SD (fl)	31.50 ± 2.1	33.40 ± 0.84	33.50 ± 2.12	32.90 ± 0.14	32.60 ± 2.12	32.80 ± 1.83	34.0 ± 1.40	33.40 ± 0.56
PLT (10^3^/μL)	1172.00 ± 2.82	1169.5 ± 1.41	1168.50 ± 4.95	1184.00 ± 5.65	941.50 ± 173.24	851.50 ± 129.40	846.0 ± 76.36	1240.50 ± 0.70
MPV (fl)	5.4 ± 0.84	6.35 ± 2.05	6.50 ± 0.70	5.40 ± 0.84	6.60 ± 1.83	7.80 ± 0.00	8.0 ± 1.41	6.50 ± 0.70
NE (10^3^/μL)	14.15 ± 3.88	12.10 ± 9.19	14.00 ± 1.41	12.35 ± 1.48	14.00 ± 9.28	12.35 ± 0.91	12.25 ± 0.63	12.90 ± 0.56
LY (10^3^/μL)	82.4 ± 0.14	83.25 ± .63	82.15 ± 0.35	80.80 ± 0.70	84.20 ± 1.83	84.75 ± 1.20	86.25 ± 1.34	84.65 ± 3.18
MO (10^3^/μL)	0.70 ± 0.14	0.60 ± 0.00	0.60 ± 0.14	0.50 ± 0.28	1.15 ± 1.20	0.30 ± 0.00	0.35 ± 0.07	0.70 ± 0.70
EO (10^3^/μL))	0.35 ± 4.03	0.05 ± 0.07	0.15 ± 0.07	1.85 ± 2.47	0.05 ± 0.07	0.100 ± 0.00	0.15 ± 0.07	0.25 ± 0.21

WBC, white blood cell; RBC, red blood cell; HGB, hemoglobin; HCT, hematocrit; MCV, mean corpuscular volume; MCH, mean corpuscular hemoglobin; RDW, red cell distribution; RDW-SD, red cell distribution width standard deviation; PLT, platelet; MPV, mean platelet volume; NE, neutrophils; LY, lymphocytes; MO, monocytes; EO, eosinophils.

The measurement values are in Mean ± SD.

Similarly, the hematological parameters were measured in male and female mice which were the two types of extracts. This included

WBC, RBC, HGB, HCT, MCV, MCH, RDW, RDW-SD, PLT, MPV, NE, LY, MO and EO. The results showed that all these parameters fell within normal ranges when compared to a control group. Overall, there were no significant differences in the average hematological parameters between the control and treated groups (Table 4).

**Table 4:** Effects of low- (125mg/kg), medium- (150mg/kg) and high-doses (500mg/kg) 70% ethanol extracts of *R. abyssinicus* fruit on hematological parameters of mice.

Parameter	Male				Female			
	Control	Extract doses			Control	Extract doses		
		Low	Medium	High		Low	Medium	High
WBC (10^3^/μL)	6.30 ± 0.56	7.00 ± 0.14	7.95 ± 1.06	7.10 ± 0.42	6.85 ± 0.21	7.00 ± 0.98	6.95 ± 1.06	6.10 ± 0.70
RBC (10^6^/μL)	9.00 ± 0.00	9.36 ± 0.50	9.00 ± 1.41	9.64 ± 0.50	9.32 ± 0.46	9.50 ± 0.70	9.00 ± 0.00	9.24 ± 0.33
HGB (g/dL)	15.20 ± 0.42	15.25 ± 0.35	14.65 ± 0.41	15.30 ± 1.41	15.80 ± 0.84	15.45 ± 0.63	15.75 ± 1.48	14.95 ± 1.20
HCT (%)	45.60 ± 1.27	50.40 ± 1.98	49.50 ± 4.95	47.20 ± 2.40	48.50 ± 0.99	44.00 ± 1.41	44.50 ± 3.53	47.45 ± 0.07
MCV(fl)	49.50 ± 0.70	50.50 ± 2.1	51.00 ± 1.41	49.55 ± 0.63	50.25 ± 1.06	53.50 ± 2.12	53.00 ± 2.82	50.05 ± 0.07
MCH (pg)	16.00 ± 1.41	15.45 ± 0.63	15.00 ± 2.82	15.70 ± 0.42	16.50 ± 0.70	17.00 ± 0.00	18.00 ± 1.41	15.45 ± 0.77
MCHC (g/dL)	31.50 ± 0.70	30.45 ± 0.77	29.50 ± 2.12	31.25 ± 0.35	31.45 ± 0.63	32.00 ± 1.41	32.50 ± 0.70	30.30 ± 0.99
RDW (%)	16.50 ± 0.70	17.60 ± 0.84	17.50 ± 2.12	17.35 ± 0.91	16.70 ± 0.42	16.00 ± 1.41	16.50 ± 2.12	16.00 ± 0.00
RDW-SD (fl)	29.50 ± 0.70	30.50 ± 0.70	31.00 ± 4.24	30.60 ± 0.56	29.90 ± 0.14	31.50 ± 0.70	32.00 ± 1.41	30.10 ± 0.14
PLT (10^3^/μL)	1100.00 ± 42.42	1125.00 ± 35.35	1139.00 ± 5.65	1125.00 ± 35.35	920.00 ± 28.28	925.00 ± 35.35	900.00 ± 28.20	970.50 ± 57.27
MPV (fl)	7.00 ± 1.41	5.55 ± 0.63	6.00 ± 1.41	7.40 ± 0.56	7.90 ± 0.14	8.70 ± 0.99	8.00 ± 1.40	6.90 ± 1.27
NE (10^3^/μL)	16.85 ± 1.20	18.40 ± 0.56	18.55 ± 0.91	18.65 ± 1.06	14.20 ± 0.84	14.50 ± 0.70	14.50 ± 0.70	13.45 ± 1.76
LY (10^3^/μL)	82.50 ± 0.70	80.00 ± 0.70	78.95 ± 1.62	84.70 ± 3.11	82.80 ± 0.84	87.05 ± 2.75	88.50 ± 2.12	85.25 ± 14.77
MO (10^3^/μL)	0.25 ± 0.07	0.35 ± 0.07	0.40 ± 0.14	0.35 ± 0.21	0.85 ± 0.77	1.45 ± 0.21	1.35 ± 0.21	0.85 ± 0.49
EO (10^3^/μL)	0.15 ± 0.07	0.05 ± 0.07	1.00 ± 1.41	0.05 ± 0.07	0.60 ± 1.41	0.25 ± 0.07	0.35 ± 0.07	0.50 ± 0.56

Note: WBC, white blood cell; RBC, red blood cell; Hb, hemoglobin; HCT, hematocrit, MCV, mean corpuscular volume; MCH, mean corpuscular hemoglobin; RDW, red cell distribution; RDW-SD, red cell distribution width standard deviation; PLT, platelet; MPV, mean platelet volume; NE, neutrophils; LY, lymphocytes; MO, monocytes; EO, eosinophils.

The measurement values provided are Mean ± standard deviation (SD).

### Serum biochemical parameters

The analysis of biochemical parameters such as ALT, AST, ALP, cholesterol, TAG, LDL, HDL, Na+, K+, Cl, urea, creatinine, glucose, sodium, potassium, and chlorine in both male and female mice treated with a 70% ethanol extract of *H. abyssinica* did not show a significant change when compared with the control groups (Table 5).

**Table 5:** Effects of low- (125mg/kg), medium- (150mg/kg) and high-doses (500mg/kg) of 70% ethanol extracts of *H. abyssinica* flower on biochemical parameters of mice.

Parameter	Male				Female			
	Control	Extract dose			Control	Extract dose		
		Low	Medium	High		Low	Medium	High
ALT(SGPT) (U/L)	22.86 ± 4.06	23.43 ± 2.85	24.86 ± 3.12	24.00 ± 1.57	20.26 ± 1.04	22.53 ± 3.27	22.3 ± 0.91	22.06 ± 2.27
AST(SGOT) (U/L)	173.20 ± 41.65	167.83 ± 16.61	168.60 ± 14.60	173.93 ± 19.92	125.36 ± 84.63	127.63 ± 41.01	128.93 ± 36.46	94.53 ± 15.80
ALP (U/L)	66.33 ± 17.21	60.67 ± 2.51	59.33 ± 5.13	70.33 ± 16.01	72.33 ± 23.35	74.67 ± 15.50	73.67 ± 11.93	75.33 ± 20.55
Cholesterol (mg/dl)	131.90 ± 9.45	137.96 ± 22.21	139.60 ± 22.08	124.66 ± 16.06	123.26 ± 17.92	110.73 ± 12.68	111.46 ± 12.17	111.06 ± 28.27
TAG (mg/dl)	154.33 ± 19.74	149.66 ± 1.52	150.96 ± 1.47	144.80 ± 42.35	111.96 ± 21.06	116.66 ± 21.86	117.03 ± 22.47	104.13 ± 13.70
LDL(mg/dl)	9.93 ± 0.51	10.86 ± 3.80	11.50 ± 3.61	8.66 ± 0.86	16.33 ± 1.53	14.00 ± 6.27	14.60 ± 6.30	11.60 ± 5.82
HDL(mg/dl)	106.46 ± 8.58	117.30 ± 19.24	116.80 ± 20.48	105.83 ± 12.43	90.00 ± 13.81	78.73 ± 8.95	79.76 ± 9.09	83.3 ± 17.6
Urea (mg/dl)	43.63 ± 4.23	49.70 ± 4.92	49.96 ± 5.65	48.96 ± 1.81	41.90 ± 1.70	43.13 ± 6.27	44.33 ± 6.10	45.00 ± 3.21
Creatinine (mg/dl)	0.12 ± 0.02	0.14 ± 0.04	0.14 ± 0.04	0.10 ± 0.01	0.16 ± 0.03	0.19 ± 0.04	0.18 ± 0.02	0.14 ± 0.00
Glucose (mg/dl)	107.63 ± 1.11	106.40 ± 2.55	107.20 ± 2.62	105.03 ± 1.61	105.56 ± 2.60	105.93 ± 5.51	105.10 ± 4.46	109.43 ± 11.85
Na+ (mmol/l)	150.33 ± 1.15	150.67 ± 1.52	149.00 ± 1.00	152.33 ± 2.08	150.67 ± 1.52	155.33 ± 2.88	152.67 ± 2.51	153.67 ± 3.05
K+ (mmol/l)	6.82 ± 1.11	6.87 ± 0.75	6.56 ± 0.60	6.38 ± 0.43	5.45 ± 0.28	6.67 ± 1,19	6.30 ± 1.03	5.63 ± 1.03
Cl^-^ (mmol/l)	108.53 ± 0.85	110.46 ± 1.20	109.20 ± 0.90	109.86 ± 1.66	111.10 ± 0.51	112.20 ± 1.37	111.00 ± 0.95	109.90 ± 1.58

ALT, alanine aminotransferase; AST, aspartate aminotransferase; ALP, alkaline phosphatase; TAG, triacylglycerol; LDL, Low-density lipoprotein; HDL, high-density lipoprotein; Na+, sodium; K+, Potassium, Cl-, chlorine.

The measurement values provided are Mean ± standard deviation (SD).

Similarly, the examination of biochemical factors like ALT, AST, ALP, cholesterol, TAG, LDL, HDL, Na+, K+, Cl in male and female mice who received a 70% ethanol extract of *R. abyssinicus* did not demonstrate a noteworthy difference when compared to the control groups (Table 6).

**Table 6:** Effects of low- (125mg/kg), medium- (150mg/kg) and high-doses (500mg/kg) of 70% ethanol extracts of *R. abyssinicus* flower extract on biochemical parameters of mice.

Parameter	Male				Female			
	Control	Extract doses			Control	Extract doses		
		Low	Medium	High		Low	Medium	High
ALT(SGPT) (U/L)	24.66 ± 0.90	24.76 ± 2.92	25.30 ± 1.27	23.75 ± 4.17	22.80 ± 5.93	22.16 ± 2.74	21.50 ± 0.55	18.96 ± 0.95
AST(SGOT) (U/L)	205.96 ± 56.22	199.96 ± 73.12	196.00 ± 61.22	228.65 ± 55.0	173.03 ± 33.39	192.76 ± 75.33	189.26 ± 71.39	99.40 ± 32.93
ALP (U/L)	93.00 ± 29.51	63.33 ± 15.94	62.33 ± 13.79	78.50 ± 10.60	67.00 ± 17.34	75.33 ± 20.20	73.33 ± 15.27	75.67 ± 13.27
Cholesterol (mg/dl)	144.46 ± 5.92	144.80 ± 8.62	140.83 ± 7.20	130.15 ± 28.35	106.30 ± 13.19	119.60 ± 8.41	114.16 ± 5.15	116.40 ± 10.95
TAG (mg/dl)	103.20 ± 28.83	114.80 ± 18.36	112.36 ± 15.28	149.40 ± 65.19	134.33 ± 33.97	116.20 ± 20.63	119.10 ± 20.33	135.10 ± 25.64
LDL(mg/dl)	12.56 ± 1.48	11.30 ± 1.64	10.43 ± 0.66	9.00 ± 6.08	11.26 ± 2.74	13.36 ± 0.65	12.80 ± 0.52	12.63 ± 3.28
HDL(mg/dl)	87.90 ± 27.72	119.86 ± 7.93	113.93 ± 5.79	105.35 ± 21.56	71.43 ± 14.01	87.20 ± 30.46	85.43 ± 28.32	78.93 ± 10.04
Urea (mg/dl)	37.76 ± 2.50	40.56 ± 2.01	39.76 ± 0.58	36.35 ± 2.33	43.60 ± 3.53	45.83 ± 4.41	45.30 ± 5.28	45.46 ± 1.45
Creatinine (mg/dl)	0.11 ± 0.01	0.14 ± 0.01	0.13 ± 0.01	0.10 ± 0.00	0.17 ± 0.06	0.14 ± 0.04	0.15 ± 0.03	0.17 ± 0.05
Glucose (mg/dl)	112.66 ± 11.85	107.33 ± 3.43	104.50 ± 3.15	107.05 ± 0.35	106.96 ± 0.81	113.23 ± 8.30	109.53 ± 5.55	108.53 ± 1.30
Na^+^ (mmol/l)	152.00 ± 2.64	153.67 ± 2.08	149.33 ± 1.15	150.50 ± 3.53	153.00 ± 3.60	150.67 ± 2.51	147.67 ± 3.21	157.00 ± 2.64
K^+^ (mmol/l)	5.87 ± 0.58	6.44 ± 0.06	6.06 ± 0.11	5.77 ± 0.10	7.31 ± 0.54	7.77 ± 1.93	7.29 ± 1.14	6.05 ± 0.67
Cl^-^ (mmol/l)	110.53 ± 4.18	112.60 ± 1.11	110.46 ± 0.55	115.80 ± 0.14	114.66 ± 0.76	113.56 ± 1.25	111.86 ± 1.69	116.73 ± 1,43

ALT, alanine aminotransferase; AST, aspartate aminotransferase; ALP, alkaline phosphatase; TAG, triacylglycerol; LDL, Low-density lipoprotein; HDL, high-density lipoprotein; Na+, sodium; K+, Potassium, Cl-, chlorine.

The measurement values are in mean ± SD.

### Histopathology

**Fig 1:**
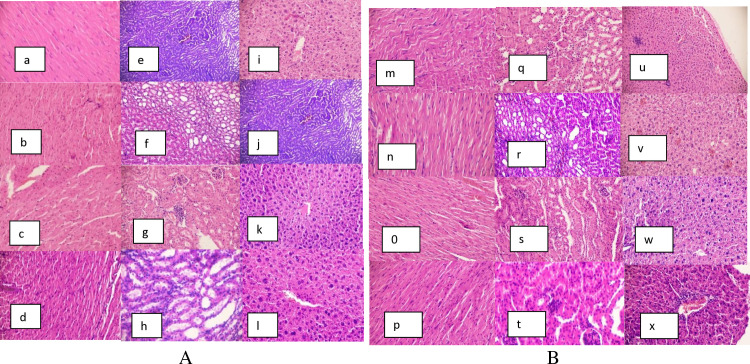
A: shows histopathological examinations of the male mice: (a) heart, (e) kidney, and (i) liver in the control group; (b) heart, (f) kidney, and (j) liver in 125 mg/kg *H. abyssinica* group; (c) heart, (g) kidney, and (k) liver in 250 mg/kg *H. abyssinica* group and (d) heart, (h) kidney, and (i) liver in 500 mg/kg *H. abyssinica* group. B: shows histopathological examinations of the female mice (m) heart, (q) kidney, and (u) liver in the control group; (n) heart, (r) kidney, and (v) liver in 125 mg/kg *H. abyssinica* group; (o) heart, (s) kidney and (w) liver in 250 mg/kg *H. abyssinica* group and (p) heart, (t) kidney, and (x) liver in 500 mg/kg *H. abyssinica* group.

The heart, kidneys, and liver exhibited normal histology and were comparable to those of the control group mice (Fig 1A and 1B).

**Fig 2:**
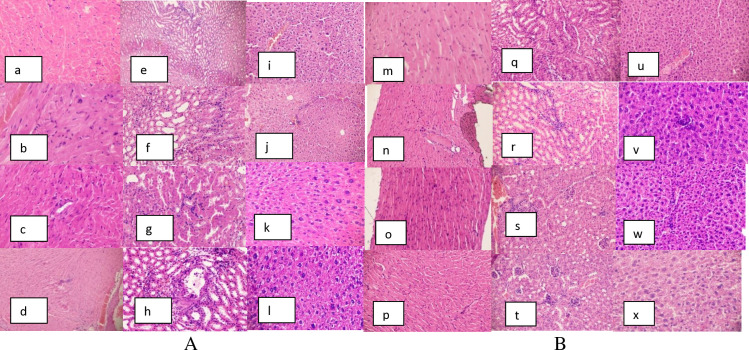
A: shows histopathological examinations of the male mice: (a) heart, (e) kidney, and (i) liver in the control group; (b) heart, (f) kidney, and (j) liver in 125 mg/kg *R. abyssinicus* group; (c) heart, (g) kidney, and (k) liver in 250 mg/kg *R. abyssinicus* group and (d), heart, (h) kidney, and (i) liver in 500 mg/kg *R. abyssinicus* group. B: shows histopathological examinations of the female mice: (m) heart, (q) kidney, and (u) liver in the control group; (n) heart, (r) kidney, and (v) liver in 125 mg/kg *R. abyssinicus* group; (o) heart, (s) kidney and (w) liver in 250 mg/kg *R. abyssinicus* group and (p) heart, (t) kidney and (x) liver in 500 mg/kg *R. abyssinicus* group.

Similarly, the heart, kidneys, and liver, of both male and female mice treated with *R. abyssinicus* extract did not reveal abnormal and were similar to that of the control group mice (Figs 2A and 2B).

## Discussion

This study was carried out to evaluate the potential acute and sub-acute toxic effects of 70% ethanolic flower extracts of *H. abyssinica* and *R. abyssinicus* on Swiss albino mice models. When mice were given the highest oral dose of 2,000 mg/kg of both the 70% ethanol flower extracts, no lethality was observed. It was found that the LD_50_ of the plant extract is greater than 2,000 mg/kg. No immediate or delayed signs of toxicity were noted. This is in line with previous research on methanol rhizome extract of *R. abyssinicus* and flower extracts of *H. abyssinica*, which also showed LD_50_ values greater than 2,000 mg/kg and 5,000 mg/kg [42,43]. Based on the OECD’s classification of toxic substances, any plant extract or compound with an oral LD_50_ greater than 1,000 mg/kg can be deemed safe and low in toxicity [44]. Therefore, the 70% ethanol extract of *H. abyssinica* and *R. abyssinicus* flower can be considered non-toxic at a single dose level of 2,000 mg/kg bodyweight in mice.

The sub-acute toxicity evaluation revealed no toxic-related illnesses or deaths following oral exposure to doses of 125, 250, or 500 mg/kg over a 4-week period. This indicates that the extracts are safe at all tested doses. This outcome is consistent with earlier research that no toxic-related illnesses or deaths were reported with methanol rhizome extract of *R. abyssinicus* and flower extracts of *H. abyssinica* [42,43]. The change in weight of animals is a crucial factor in assessing their overall health and welfare [45]. During the treatment period, mice administered with the extracts displayed continual growth in bodyweight; however, this increase was not considered statistically significant when compared to the control group implying that the treatments did not influence their appetite, food intake, or natural fluctuations in bodyweight.

The Society of Toxicologic Pathology (STP) suggests that organ weight should be incorporated into toxicity studies to evaluate treatment-related impacts [38]. The kidneys, liver, and heart, are the most frequently assessed organs for this purpose. Alterations in liver weight may signal treatment-related effects such as hepatocellular hypertrophy [46]. Likewise, changes in heart and kidney weight could denote treatment-related effects like renal toxicity, tubular hypertrophy, or chronic progressive nephropathy [38]. In the current study, it was found that the relative organ weight of female and male mice treated with the extracts did not significantly increase compared to their respective control groups. This indicates that the extracts were not toxic to the animals at the given doses.

Hematological parameters are assessed to determine the impact of plant extracts on experimental animals. Toxic doses can cause changes in blood parameters indicating hematological disorders [47]. However, this study found no significant differences in all hematological parameters between male and female mice treated with the two plant extracts at different doses, and their respective control. This finding is consistent with similar studies that used methanolic rhizome extracts of *R. abyssinicus* and aqueous extracts of *H. abyssinica* flowers, which found no changes in the hematological parameters count when compared with the control groups [42,43]. Therefore, it suggests that the 70% ethanol extract of both plants is safe at the studied doses in mice.

The levels of certain biochemical parameters are used to evaluate the functioning of the liver and kidneys [48]. The kidneys are particularly at risk from toxic substances due to their high rate of blood perfusion and their ability to concentrate various substances in the tubular lumen [49]. Changes in serum creatinine, urea, and electrolyte levels like sodium, potassium, chloride, and magnesium can indicate kidney issues [50]. After administering 70% ethanolic flower extracts of *H. abyssinica* and *R. abyssinica*, there was no significant increase in the level of creatinine in mice treated with 125 mg/kg, 250 mg/kg, and 500 mg/kg compared to the control. Similarly, urea levels did not significantly increase in treated with the same doses compared to the control. The concentration of sodium, potassium, and chloride ions was not significantly affected by the extract administration at any of the doses in this study.

Similarly, changes in ALT, AST, and glucose levels may indicate liver damage [51]. ALT and AST serum levels increasing indicate the extent of the tissue damage [52]. The current finding showed that there was no significance difference in the levels of ALT and AST, between male and female mice in the three dosage groups that received a 70% ethanol extract of *H. abyssinica* and *R. abyssinicus* flower, in comparison to the control group.

Changes in the lipid profile levels during serum biochemical parameter analysis indicate various health conditions [53]. However, when Swisss albino mice were administered the extracts of at doses of 125 mg/kg, 250 mg/kg, and 500 mg/kg, no significant difference was observed in the levels of cholesterol, TAG, LDL and, HDL compared to the control group.

Histopathological examinations offer additional insights to support the findings from biochemical and hematological analyses. The current histological examination revealed that the heart, kidney, and liver of mice treated with 70% ethanol extracts of *H. abyssinica* and *R. abyssinicus* displayed normal histology, comparable to the control group. These results align with the findings of Fentahun *et al.* (2020) and Kimmo (2005), who observed similar outcomes when evaluating the histopathology of mice organs treated with methanol rhizome extract of *R. abyssinicus* and flower extracts of *H. abyssinica* [42,43].

The biochemical, hematological, and histopathological analyses in this study suggest that the flower extracts of *R. abyssinicus* and *H. abyssinica* are safe for use. Previous phytochemical analyses have indicated the presence of flavonoids, terpenoids, and phenols in both *R. abyssinicus* and *H. abyssinica* extracts [28,54]. These compounds may significantly contribute to their safety profile. For instance, the flavonoids and phenols found in the extracts could help prevent oxidative damage by scavenging free radicals and chelating metal ions, thereby protecting cells from oxidative stress [55–58]. This oxidative stress can negatively impact various cellular structures, including membranes, lipids, proteins, lipoproteins, and deoxyribonucleic acid (DNA) [59–63]. Similarly, terpenoids, are widely recognized for their antimicrobial, anti-inflammatory, and antioxidant properties [64]. Their presence could enhance the detoxification processes [65].

The main limitations of the current study include a narrow dose range, a short duration, and a minimum sample size (3 mice per group). The acute toxicity study evaluated only a single high dose (2,000 mg/kg), while the sub-acute study assessed only three levels (125, 250, and 500 mg/kg). A broader dose range, including both lower and higher doses, is essential for understanding the dose-response relationship and potential toxicity thresholds. Additionally, the short duration of the sub-acute study may not capture the effects of prolonged exposure, necessitating longer studies to identify any delayed or cumulative toxic effects.

Future studies should explore dose ranges that prioritize chronic toxicity assessment, as well as reproductive and developmental toxicity. Additionally, a recovery group should be included to examine the persistence or delayed occurrence of toxic effects. By addressing these key endpoints, it is possible to develop a thorough understanding of the safety and potential risks associated with *H. abyssinica* and *R. abyssinicus*, facilitating their safe use in therapeutic and dietary contexts, as well as in aquatic ecosystems against schistosomiasis intermediate hosts.

Given the current findings, it would not be appropriate to conclude that *H. abyssinica* and *R. abyssinicus* extracts are safe for use in aquatic environments against schistosome intermediate hosts without further studies. The limited scope of the toxicity studies, which were conducted in rodents, does not address the potential toxicity of the extracts in aquatic environments where the exposure route and target organisms differ. Additionally, no information is provided on the toxicity to aquatic organisms, necessitating dedicated studies to evaluate the extracts’ aquatic toxicity profile. Understanding the persistence and behavior of the extracts in aquatic ecosystems is crucial for predicting potential harm to non-target organisms over time. Moreover, demonstrating selective toxicity towards the target schistosome intermediate hosts, compared to other non-target aquatic species, is essential for the safe use of these extracts.

## Conclusion

The findings from this study indicate that the 70% ethanol extracts of *H. abyssinica* and *R. abyssinicus* flowers exhibited no visible or measurable toxicity in the tested animal model. The acute toxicity evaluation revealed that the LD_50_ of both plant extracts exceeds 2000 mg/kg bodyweight, which is classified as having a low toxicity profile according to international guidelines. Sub-acute toxicity assessment further confirmed the absence of significant toxicity. Oral administration of the extracts at doses up to 500 mg/kg for four weeks did not lead to any treatment-related illnesses, fatalities, or noteworthy changes in bodyweight, organ weights, hematological parameters, or clinical biochemistry in Swiss albino mice. Histopathological examinations of major organs showed no abnormal histology compared to the control group. Future studies should employ larger sample sizes with broader dose ranges, prioritizing assessments of chronic toxicity, reproductive, and developmental toxicity. Addressing these key endpoints will provide a comprehensive understanding of the safety and potential risks associated with the plants, facilitating their safe use in therapeutic, dietary and molluscicidal contexts. In particular, further studies evaluating the toxicity of the extracts to various relevant aquatic organisms, as well as their environmental fate and behavior, are essential to prevent significant threats to non-target species when used as molluscicides. Studies focused solely on rodent toxicity do not directly qualify these plants for field application. Overall, the preliminary findings partly support the longstanding traditional medicinal value of these plants, suggesting their safety for community use.

## Supporting information

S1 File The dataset encompasses hematological and biochemical parameters, as well as body weight measurements of the mice. (XLXS)

## References

[1] Sturrock RF. The schistosomes and their intermediate hosts. In: Mahmoud AAF, editor. Schistosomiasis. London: Imperial College Press; 2001.

[2] Coon DR. Schistosomiasis: overview of the history, biology, clinicopathology, and laboratory diagnosis. Clin Microbiol Newsl. 2005;27(21):163–8. doi: 10.1016/j.clinmicnews.2005.10.001

[3] Zoni AC, Catala L, Ault SK. Schistosomiasis prevalence and intensity of infection in Latin America and the Caribbean countries, 1942-2014: a systematic review in the context of a regional elimination goal. PLoS NeglTrop Dis. 2016;10(3):e0004493. doi: 10.1371/journal.pntd.0004493 27007193 PMC4805296

[4] Aula OP, McManus DP, Jones MK, Gordon CA. Schistosomiasis with a focus on Africa. Trop Med Infect Dis. 2021;6(3):109. doi: 10.3390/tropicalmed6030109 34206495 PMC8293433

[5] World Health Organization. Schistosomiasis and soil-transmitted helminthiases: progress report, 2022. Wkly Epidemiol Rec. 2023;98(51):667–76. [cited 15 Sep 2024]. Available from: https://www.who.int/publications/i/item/who-wer9851-667-676

[6] Ethiopia Ministry of Health. The Third National Neglected Tropical Diseases Strategic Plan 2021-2025. Addis Ababa: Ministry Health of Ethiopia; 2021. [cited 15 Sep 2024]. Available from: https://espen.afro.who.int/system/files/content/resources/Third%20NTD%20national%20Strategic%20Plan%202021-2025.pdf

[7] Kloos H, Lo CT, Birrie H, Ayele T, Tedla S, Tsegay F. Schistosomiasis in Ethiopia. Soc Sci Med. 1988;26(8):803–27. doi: 10.1016/0277-9536(88)90174-8 3131881

[8] Woldeyohannes D, Sahiledengle B, Tekalegn Y, Hailemariam Z. Prevalence of Schistosomiasis (S. mansoni and S. haematobium) and its association with gender of school age children in Ethiopia: A systematic review and meta-analysis. Parasite Epidemiol Control. 2021;13:e00210. doi: 10.1016/j.parepi.2021.e00210 33842698 PMC8020476

[9] World Health Organization. Ending the neglect to attain the Sustainable Development Goals: a road map for neglected tropical diseases 2021–2030. WHO, Geneva 2021. [cited 15 Sep 2024]. Available from: https://www.who.int/publications/i/item/9789240010352

[10] Vale N, Gouveia MJ, Rinaldi G, Brindley PJ, Gärtner F, da Costa JMC. Praziquantel for schistosomiasis: single-drug metabolism revisited, mode of action, and resistance. Antimicrob Agents Chemother. 2017;61(5): doi: 10.1128/aac.02582-16 PMC5404606 28264841

[11] Inobaya MT, Olveda RM, Chau TN, Olveda DU, Ross AG. Prevention and control of schistosomiasis: a current perspective. Res Rep Trop Med. 2014;2014(5):65–75. doi: 10.2147/RRTM.S44274 25400499 PMC4231879

[12] Stelma F, Talla I, Sow S, Kongs A, Niang M, Polman K, et al. Efficacy and side effects of praziquantel in an epidemic focus of *Schistosoma mansoni*. Am J Trop Med Hyg. 1995;53(2):167–70. doi: 10.4269/ajtmh.1995.53.167 7677219

[13] Pica-Mattoccia L, Cioli D. Sex-and stage-related sensitivity of *Schistosoma mansoni* to in vivo and in vitro praziquantel treatment. Int J Parasitol. 2004;34(4):527–33. doi: 10.1016/j.ijpara.2003.12.003 15013742

[14] Ismail MM, Farghaly AM, Dyab AK, Afify HA, el-Shafei MA. Resistance to praziquantel, effect of drug pressure and stability test. J Egypt Soc Parasitol. 2002;32(2):589–600. 12214936

[15] Silva LM, Menezes RM, de Oliveira SA, Andrade ZA. Chemotherapeutic effects on larval stages of *Schistosoma mansoni* during infection and re-infection of mice. Rev Soc Bras Med Trop. 2003;36(3):335–41. doi: 10.1590/s0037-86822003000300004 12908033

[16] Grimes JE, Croll D, Harrison WE, Utzinger J, Freeman MC, Templeton MR. The roles of water, sanitation and hygiene in reducing schistosomiasis: a review Parasit Vectors. 2015;8(1):1–16. doi: 10.1186/s13071-015-0766-9 25884172 PMC4377019

[17] Feng J, Wang X, Zhang X, Hu H, Xue J, Cao C, et al. Effect of health education on schistosomiasis control knowledge, attitude, and practice after schistosomiasis blocking: results of a longitudinal observational study in the field. Trop Med Infect Dis. 2023;8(5):267. doi: 10.3390/tropicalmed8050267 37235315 PMC10222372

[18] Lo NC, Gurarie D, Yoon N, Coulibaly JT, Bendavid E, Andrews JR, et al. Impact and cost-effectiveness of snail control to achieve disease control targets for *schistosomiasis*. Proc Natl Acad Sci USA. 2018;115(4):E584–91. doi: 10.1073/pnas.1708729114 29301964 PMC5789907

[19] Marston A, Hostettmann K. Review article number 6: Plant molluscicides. Phytochem. 1985;24(4):639–52. doi: 10.1016/s0031-9422(00)84870-0

[20] Andrews P, Thyssen J, Lorke D. The biology and toxicology of molluscicides, Bayluscide. Pharmacol Ther. 1982;19(2):245–95. doi: 10.1016/0163-7258(82)90064-x 6763710

[21] Coelho PM, Caldeira RL. Critical analysis of molluscicide application in *schistosomiasis* control programs in Brazil. Infect. Dis. Poverty. 2016;5(1):57. doi: 10.1186/s40249-016-0153-6 27374126 PMC4931695

[22] Pieri OS, Gonçalves JF, Sarquis O. Repeated focal mollusciciding for snail control in a sugar-cane area of northeast Brazil. Mem Inst Oswaldo Cruz. 1995;90(4):535–6. doi: 10.1590/s0074-02761995000400022 8551961

[23] Amsalu N, Bezie Y, Fentahun M, Alemayehu A, Amsalu G. Use and conservation of medicinal plants by indigenous people of Gozamin Wereda, East Gojjam Zone of Amhara region, Ethiopia: an ethnobotanical approach. Evid Based Complement Alternat Med. 2018;2018(1):2973513. doi: 10.1155/2018/2973513 29743921 PMC5884302

[24] Assefa B, Glatzel G, Buchmann C. Ethnomedicinal uses of Hagenia abyssinica (Bruce) JF Gmel. among rural communities of Ethiopia. J Ethnobiol Ethnomed. 2010;6(1):20. doi: 10.1186/1746-4269-6-20 20701760 PMC2928183

[25] Plants of the World. Hagenia abyssinica (Bruce) J.F.Gmel., Syst. Nat., ed. 13[bis]: 613 (1791). [cited 15 Sep 2024]. Available from: https://powo.science.kew.org/taxon/urn:lsid:ipni.org:names:725448-1#publications

[26] Plants of the World. Rumex abyssinicus Jacq., Hort. Bot. Vindob., 3: 48 (1777). [cited 15 Sep 2024]. Available from: https://powo.science.kew.org/taxon/urn:lsid:ipni.org:names:696816-1

[27] Mulisa E, Asres K, Engidawork E. Evaluation of wound healing and anti-inflammatory activity of the rhizomes of *Rumex abyssinicus* J.(Polygonaceae) in mice. BMC Complement Altern Med. 2015;15(1):1–10. doi: 10.1186/s12906-015-0878-y 26423525 PMC4589968

[28] Nigussie G, Tola M, Fanta T. Medicinal uses, chemical constituents and biological activities of Rumex abyssinicus: a Comprehensive review. Int J Second Metab. 2022;9(4):440–56. doi: 10.21448/ijsm.1095643

[29] Basha H, Debella A, Endale M, Debebe E, Mathewos M, Biftu T, et al. Molluscicidal activity of extracts and fractions from Hagenia abyssinica, Rosa abyssinica, and Cucumis ficifolius against biomphalaria and bulinus snails. J Parasitol Res. 2024;2024(1):7968654. doi: 10.1155/japr/7968654 39624255 PMC11611416

[30] Bandiola Teresa May. Extraction and qualitative phytochemical screening of medicinal plants: a brief summary. Int J Pharm. 2018;8(1):137–43. [cited 15 Sep 2024]. Available from: https://www.researchgate.net/publication/324674203_Extraction_and_Qualitative_Phytochemical_Screening_of_Medicinal_Plants_A_Brief_Summary

[31] National Research Council. Guide for the care and use of laboratory animals: Eighth Edition. Washington, DC: The National Academies Press; 2011. [cited 15 Sep 2024]. Available from: https://grants.nih.gov/grants/olaw/guide-for-the-care-and-use-of-laboratory-animals.pdf

[32] European Union (EU). Directive 2010/63/EU of the European Parliament and of the Council of 22 September 2010 on the protection of animals used for scientific purposes. [cited 15 Sep 2024]. Available from: https://eur-lex.europa.eu/LexUriServ/LexUriServ.do?uri=OJ:L:2010:276:0033:0079:en:PDF

[33] American Veterinary Medical Association. Guidelines for the Euthanasia of animals: 2020 edition [cited 15 Sep 2024]. Available from: https://www.avma.org/sites/default/files/2020-02/Guidelines-on-Euthanasia-2020.pdf

[34] Laferriere CA, Pang DS. Review of intraperitoneal injection of sodium pentobarbital as a method of euthanasia in laboratory rodents. J Am Assoc Lab Anim Sci. 59(3):254–63. doi: 10.30802/AALAS-JAALAS-19-000081 PMC7210732 32156325

[35] Du Sert NP, Ahluwalia A, Alam S, Avey MT, Baker M, Browne WJ, et al. Reporting animal research: explanation and elaboration for the ARRIVE guidelines 2.0. PLoS Biol. 2020;18(7):e3000411. doi: 10.1371/journal.pbio.3000411 32663221 PMC7360025

[36] Organization for Economic Co-operation and Development. Test No. 423: Acute Oral toxicity - Acute Toxic Class Method, OECD Guidelines for the Testing of Chemicals, Section 4, OECD Publishing; 2001. [cited 15 Sep 2024]. Available from: https://ntp.niehs.nih.gov/sites/default/files/iccvam/suppdocs/feddocs/oecd/oecd_gl423.pdf

[37] Organisation for Economic Co-operation and Development. Test no. 407: repeated dose 28-day oral toxicity study in rodents. OECD Publishing; 2008. [cited 15 Sep 2024]. Available from: https://www.oecd-ilibrary.org/environment/test-no-407-repeated-dose-28-day-oral-toxicity-study-in-rodents_9789264070684-en

[38] Sellers RS, Morton D, Michael B, Roome N, Johnson JK, Yano BL, et al. Society of Toxicologic Pathology position paper: organ weight recommendations for toxicology studies. Toxicol Pathol. 2007;35(5):751–5. doi: 10.1080/01926230701595300 17849358

[39] Das N, Goshwami D, Hasan MS, Raihan SZ. Evaluation of acute and subacute toxicity induced by methanol extract of Terminalia citrina leaves in Sprague Dawley rats. J Acute Dis. 2015;4(4):316–21. doi: 10.1016/j.joad.2015.05.001

[40] Deyno S, Tola MA, Bazira J, Makonnen E, Alele PE. Acute and repeated-dose toxicity of *Echinops kebericho* Mesfin essential oil. Toxicol Rep. 2021;8:131–8. doi: 10.1016/j.toxrep.2020.12.027 33437654 PMC7787995

[41] Slaoui M, Fiette L. Histopathology procedures: from tissue sampling to histopathological evaluation. Methods Mol Biol. 2011;691:69–82. doi: 10.1007/978-1-60761-849-2_4 20972747

[42] Kimmo JD. Toxicological Study of Glinus lotoides and Hagenia abyssinica: traditionally used taenicidal herbs in Ethiopia (Doctoral dissertation, Addis Ababa University); 2005 Available from: http://thesisbank.jhia.ac.ke/id/eprint/6608

[43] Fentahun E, Muche A, Endale A, Tenaw B. Evaluation of the acute and sub-acute toxic effects of 80% methanol rhizome extracts of Rumex abyssinicus jacq. (Plygonaceae) on histopathology of liver, kidney and some blood parameters in Swiss Albino Mice. ECPT. 2020;8(7):01–12. Available from: https://www.researchgate.net/publication/343813293

[44] Adeneye AA, Olagunju JA. Preliminary hypoglycemic and hypolipidemic activities of the aqueous seed extract of Carica papaya Linn in Wistar rats. Biol Med. 2009;1(1):1–10. Available from: https://citeseerx.ist.psu.edu/document?repid=rep1&type=pdf&doi=73e4d5418836ccf0f505bf055b694e4fdc2dd81f

[45] Unuofin JO, Otunola GA, Afolayan AJ. Evaluation of acute and subacute toxicity of whole-plant aqueous extract of *Vernonia mespilifolia* Less. in Wistar rats. J Integr Med. 2018;16(5):335–41. doi: 10.1016/j.joim.2018.07.003 30007829

[46] Hall AP, Elcombe CR, Foster JR, Harada T, Kaufmann W, Knippel A, et al. Liver hypertrophy: a review of adaptive (adverse and non-adverse) changes—conclusions from the 3rd International ESTP Expert Workshop. Toxicol Pathol. 2012;40(7):971–94. doi: 10.1177/0192623312448935 22723046

[47] Arika WM, Nyamai DW, Musila MN, Ngugi MP, Njagi ENM. Hematological markers of in vivo toxicity. J Hematol Thrombo Dis. 2016;4:236. doi: 10.4172/2329-8790.1000236

[48] Husic-Selimovic A, Medjedovic S, Bijedic N, Sofic A. Biochemical parameters as predictors of underlying liver disease in patients with chronic kidney disorders. Acta Inform Med. 2021;29(4):260–5. doi: 10.5455/aim.2021.29.260-265 35197660 PMC8800580

[49] Makris K, Spanou L. Acute kidney injury: definition, pathophysiology, and clinical phenotypes. Clin Biochem Rev. 2016;37(2):85–98. doi: 10.1093/ckj/sfaa142 28303073 PMC5198510

[50] Salazar JH. Overview of urea and creatinine. Lab. Med. 2014;45(1):e19–20. doi: 10.1309/LM920SBNZPJRJGUT

[51] Thapa B, Walia A. “Liver function tests and their interpretation”. Indian J Pediatr. 2007;74(7):663–71. doi: 10.1007/s12098-007-0118-7 17699976

[52] Huang XJ, Choi YK, Im HS, Yarimaga O, Yoon E, Kim HS. Aspartate aminotransferase (AST/GOT) and alanine aminotransferase (ALT/GPT) detection techniques. Sensors. 2006;6(7):756–82. doi: 10.3390/s6070756

[53] Castela Forte J, Gannamani R, Folkertsma P, Kumaraswamy S, Mount S, Van Dam S, et al. Changes in blood lipid levels after a digitally enabled cardiometabolic preventive health program: pre-post study in an adult Dutch general population cohort. JMIR Cardio. 2022;6(1):e34946. doi: 10.2196/34946 35319473 PMC8987960

[54] Fan M, Chen G, Zhang Y, Nahar L, Sarker SD, Hu G, et al. Antioxidant and anti-proliferative properties of Hagenia abyssinica roots and their potentially active components. Antioxidants. 2020;9(2):143. doi: 10.3390/antiox9020143 32041310 PMC7070924

[55] Othman A, Mukhtar NJ, Ismail NS, Chang SK. Phenolics, flavonoids content and antioxidant activities of 4 Malaysian herbal plants. Int. Food Res. J. 2014;21(2):759. Available from: https://www.cabidigitallibrary.org/doi/pdf/10.5555/20143367093

[56] Tungmunnithum D, Thongboonyou A, Pholboon A, Yangsabai A. Flavonoids and other phenolic compounds from medicinal plants for pharmaceutical and medical aspects: An overview. Medicines. 2018;5(3):93. doi: 10.3390/medicines5030093 30149600 PMC6165118

[57] Babbar N, Oberoi HS, Sandhu SK. Therapeutic and nutraceutical potential of bioactive compounds extracted from fruit residues. Crit Rev Food Sci Nutr. 2015;55(3):319–37. doi: 10.1080/10408398.2011.653734 24915390

[58] Aryal S, Baniya MK, Danekhu K, Kunwar P, Gurung R, Koirala N. Total phenolic content, flavonoid content and antioxidant potential of wild vegetables from Western Nepal. Plants. 2019;8(4):96. doi: 10.3390/plants8040096 30978964 PMC6524357

[59] Pizzino G, Irrera N, Cucinotta M, Pallio G, Mannino F, Arcoraci V, et al. Oxidative stress: harms and benefits for human health. Oxid Med Cell Longevity. 2017;2017(1):8416763. doi: 10.1155/2017/8416763 28819546 PMC5551541

[60] Dröge W. Free radicals in the physiological control of cell function. Physiol Rev. 2002;82(1):47–95. doi: 10.1152/physrev.00018.2001 11773609

[61] Willcox JK, Ash SL, Catignani GL. Antioxidants and prevention of chronic disease. Crit Rev Food Sci Nutr. Jul;44(4):275–95. doi: 10.1080/10408690490468489 15462130

[62] Genestra M. Oxyl radicals, redox-sensitive signalling cascades and antioxidants. Cell Signal. 2007;19(9):1807–19. doi: 10.1016/j.cellsig.2007.04.009 17570640

[63] Halliwell B. Biochemistry of oxidative stress. Biochem Soc Trans. 2007;35(Pt 5):1147–50. doi: 10.1042/BST0351147 17956298

[64] Câmara JS, Perestrelo R, Ferreira R, Berenguer CV, Pereira JA, Castilho PC. Plant-derived terpenoids: a plethora of bioactive compounds with several health functions and industrial applications—a comprehensive overview. Molecules. 2024;29(16):3861. doi: 10.3390/molecules29163861 39202940 PMC11357518

[65] Bjørklund G, Cruz-Martins N, Goh BH, Mykhailenko O, Lysiuk R, Shanaida M, et al. Medicinal plant-derived phytochemicals in detoxification. Curr Pharm Des. 2024;30(13):988–1015. doi: 10.2174/1381612829666230809094242 37559241

